# Physical and biological impacts of collimator‐scattered protons in spot‐scanning proton therapy

**DOI:** 10.1002/acm2.12653

**Published:** 2019-06-24

**Authors:** Koki Ueno, Taeko Matsuura, Shusuke Hirayama, Seishin Takao, Hideaki Ueda, Yuto Matsuo, Takaaki Yoshimura, Kikuo Umegaki

**Affiliations:** ^1^ Graduate School of Biomedical Science and Engineering Hokkaido University Sapporo Hokkaido Japan; ^2^ Faculty of Engineering Hokkaido University Sapporo Hokkaido Japan; ^3^ Proton Beam Therapy Center Hokkaido University Hospital Sapporo Hokkaido Japan; ^4^ Global Station for Quantum Medical Science and Engineering, Global Institution for Collaborative Research and Education (GI‐CoRE) Hokkaido University Sapporo Japan

**Keywords:** collimator scattering, linear energy transfer, pencil beam scanning proton therapy, relative biological effectiveness

## Abstract

To improve the penumbra of low‐energy beams used in spot‐scanning proton therapy, various collimation systems have been proposed and used in clinics. In this paper, focused on patient‐specific brass collimators, the collimator‐scattered protons' physical and biological effects were investigated. The Geant4 Monte Carlo code was used to model the collimators mounted on the scanning nozzle of the Hokkaido University Hospital. A systematic survey was performed in water phantom with various‐sized rectangular targets; range (5–20 cm), spread‐out Bragg peak (SOBP) (5–10 cm), and field size (2 × 2–16 × 16 cm^2^). It revealed that both the range and SOBP dependences of the physical dose increase had similar trends to passive scattering methods, that is, it increased largely with the range and slightly with the SOBP. The physical impact was maximized at the surface (3%–22% for the tested geometries) and decreased with depth. In contrast, the field size (FS) dependence differed from that observed in passive scattering: the increase was high for both small and large FSs. This may be attributed to the different phase‐space shapes at the target boundary between the two dose delivery methods. Next, the biological impact was estimated based on the increase in dose‐averaged linear energy transfer (*LET*_d_) and relative biological effectiveness (*RBE*). The* LET*_d_ of the collimator‐scattered protons were several keV/*μ*m higher than that of unscattered ones; however, since this large increase was observed only at the positions receiving a small scattered dose, the overall *LET*_d_ increase was negligible. As a consequence, the *RBE* increase did not exceed 0.05. Finally, the effects on patient geometries were estimated by testing two patient plans, and a negligible *RBE* increase (0.9% at most in the critical organs at surface) was observed in both cases. Therefore, the impact of collimator‐scattered protons is almost entirely attributed to the physical dose increase, while the *RBE* increase is negligible.

## 1 INTRODUCTION

Most newly built proton therapy centers worldwide are implementing the pencil beam scanning (PBS) technique because of its distinct advantages of dose conformity to targets and neutron exposure reduction compared to the more conventional passive scattering methods. However, when shallow tumors are involved, the large spot size of the low‐energy proton beam resulting from the large‐angle Coulomb scattering might offset the dose conformity advantage gained by the scanning approach.^1^ To overcome this problem, different collimators have been designed and increasingly used in the clinics^2^, ^3^, ^4^ and their clinical benefits have been investigated in various studies, with positive results.^5^

So far, most of the analytical dose calculation engines installed in the PBS treatment planning systems (TPSs) assume the collimator absorbs all the incident protons; that is, they neglect the dose contamination from the protons scattered by the collimator edge, while several studies have proven it could have a considerable dosimetric impact in reality. Van Luijk et al.^6^ used a 160‐MeV proton beam to measure and simulate scatter protons for a small field (<2 cm), showing that this contribution can be up to 20% at the patient surface. Titt et al.^7^ conducted a systematic Monte Carlo study with various target sizes and depths used in the clinics; they also suggested there is contamination from scattered protons. However, both studies focused on the scattering approach and did not systematically investigate the collimators used in PBS.

Collimator‐scattered protons can have an additional biological impact. The protons lose energy when hitting the collimator, and their linear energy transfer (LET) increases accordingly. A higher proton LET generates a greater biological effect, as observed in both *in vivo* and *in vitro* experiments^8^, ^9^, ^10^, ^11^, ^12^ and even in the clinical outcome.^13^ In the previously mentioned study, van Luijk et al.^6^ estimated the increase of the biological damage based on a simple assumption that the protons with energy above 40 MeV have a relative biological effectiveness (RBE) of 1, while for those below 40 MeV have RBE of 1.2. In their experimental setup, only 5% of protons have the energy below 40 MeV at just below the collimator, implying that the biological damage to tissues would be at most 1% larger than that expected from the physical dose. Followed by the recent rapid progress of biophysical RBE models,^8^, ^14^ it may be interesting to revisit the biological impact of scattered protons.

In this study, we investigated the physical and biological impacts of the collimator‐scattered protons used in the PBS system of the Hokkaido University Hospital. We systematically evaluated the effects of the collimator‐scattered protons on the physical dose, the dose‐averaged LET (*LET*_d_), and the RBE. To evaluate the effects in real clinical settings, we simulated two patient plans. Finally, the difference between the PBS and passive scattering systems were discussed.

## 2 MATERIALS AND METHODS

### 2.A Beam collimation system for the spot‐scanning beamline

**Figure 1:**
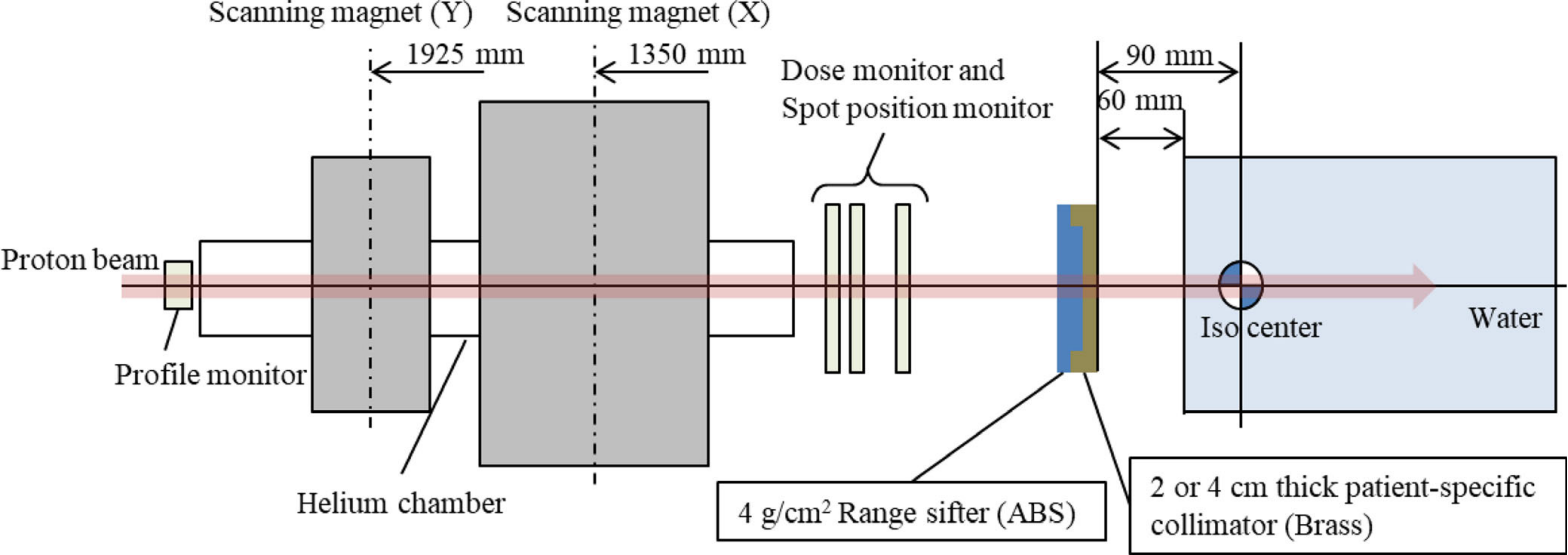
Schematic representation of the treatment nozzle with the modeled beamline and water phantom. The range sifter was made of acrylonitrile–butadiene–styrene (ABS).

The beam collimation system used for the spot‐scanning beamline simulated in this study was an extrapolation of a short‐range applicator (SRA) investigated by Yasui et al.^3^ and consisted of a 2‐ or 4‐cm thick brass collimator and a 4‐cm thick energy absorber made of acrylonitrile–butadiene–styrene (ABS) plastic (Fig. 1). In the following paragraphs, it will be referred to simply as SRA. It was mounted at the most downstream portion of the gantry, with the lower surface of the collimator placed at 9 cm from the isocenter, and designed to have a maximum uniform field of 20 × 20 cm^2^ at the isocenter. When using the configuration with the 2‐cm thick collimator, the minimum and maximum available proton ranges after passing through a 4‐cm thick energy absorber were 0.5 cm (74.9 MeV) and 10 cm (142.5 MeV) in water, respectively, and the in‐air spot size at the isocenter ranged from 11.7 to 6.0 mm (one sigma). When using the 4‐cm thick collimator, the minimum and maximum proton ranges were 0.5 cm (74.9 MeV) and 20 cm (192.4 MeV) in water, respectively, and the in‐air spot size ranged from 11.7 to 5.3 mm (one sigma).

### 2.B Simulations

#### 2.B.1 Monte Carlo code

**Figure 2:**
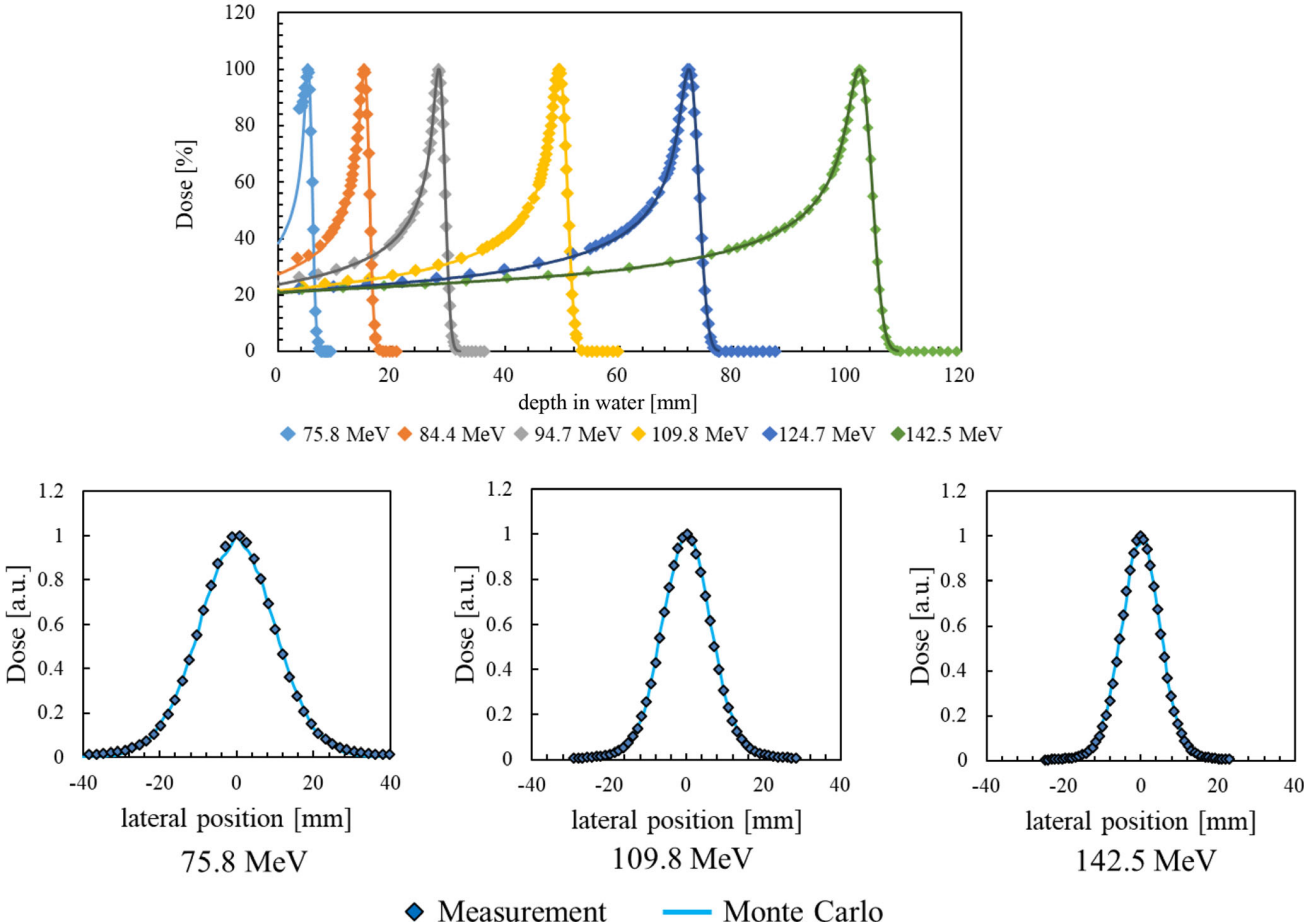
Integral depth dose (upper row) and lateral profile at a 3‐mm (for 75.8 MeV) and 5‐mm (for 109.8 and 142.5 MeV) depth in water (lower row).

The Geant4 Monte Carlo code (ver.4.10.p01)^15^ was used together with the Particle‐Therapy Simulation Framework (PTSim),^16^ a wrapper of the Geant4 toolkit that facilitates particle‐therapy simulation, to calculate the dose and *LET*_d_ distributions. The scanning nozzle of the Hokkaido University Proton Beam Therapy Centre and the SRA were modeled with different collimator opening shapes. The electromagnetic, hadron elastic and inelastic interactions were respectively simulated with the G4EmStandardPhysics option3, G4HadronElasticPhysics, and G4HadronPhysicsFTF BIC classes. The production threshold for all secondary particles was set to 1 mm. The initial beam parameters in the Monte Carlo code (i.e., Twiss parameters, emittance, mean energy, and energy dispersion) were selected to reproduce the integral depth dose and lateral beam profile in water for the nozzle with the energy absorber. Some of the validation results, up to the range of 10 cm, are illustrated in Fig. 2 and the supplementary material. In the Monte Carlo simulation, the number of protons per field was changed from 0.5 × 10^8^ to 20 × 10^8^ and their distribution in each spot depended on their intensity. The voxel size was set to 1 × 1 × 1 mm^3^.

#### 2.B.2 Target geometry

To systematically investigate the physical dose and *RBE* increase caused by the collimator‐scattered protons, the simulation was performed for different target geometries in a water tank, and their parameters are summarized in Table 1 with the corresponding abbreviations. For the target maximum range below and above 10 cm, the SRA with the 2‐ and the 4‐cm thick collimator respectively was used for the simulation. Below, we refer R[*L*_1_]_S[*L*_2_]_FS[*L*_3_] to the target with range of *L*_1_ [cm], SOBP width of *L*_2_ [cm], and FS of *L*_3_ × *L*_3_ [cm^2^]. The water surface was set 6 cm downstream of the collimator with the isocenter located at 3 cm depth in water (Fig. 1). The physical dose to the target was 2 Gy. The collimator opening size was set so to cover the edge of the proximal front of the targets with 50% of the prescribed dose.

**Table 1:** Target geometry parameters used for the simulation

Range (R) [cm]	5, 10, 15, 20
spread‐out Bragg‐ peak (SOBP) (S) [cm]	5, 10
Field size (FS) [cm^2^]	2 × 2, 4 × 4, 8 × 8, 16 × 16

#### 2.B.3 Physical dose and *LET*_d_ calculations

The physical dose and *LET*_d_ distributions were computed for collimator‐scattered protons, unscattered protons, and both together. In the dose computation, the energy within each voxel was scored at each step for all particles and events, and the total sum was converted into the physical dose. In the *LET*_d_ computation, the primary, secondary, and higher order protons were included, while the hadrons, leptons, and neutral particles generated via nuclear reactions were excluded.^17^ This was primarily because the estimated contributions of these other particles to the dose are much smaller than the protons (<1%), and they have large uncertainties for the dependence of biological parameters, that is, *α* and *β*, on *LET*_d_.^17^ For the computation of *LET*_d_ using Geant4, it has been discussed that the value changes among different scoring techniques, as well as the tracking step size limit.^18^, ^19^, ^20^ In this study, we followed Cortés‐Giraldo et al.^20^ and used the following equation for computing *LET*_d_:

(1) $$LET_{d}=\frac{\sum_{n=1}^{N}\sum_{s=1}^{S_{n}}L_{\mathrm{sn}}\varepsilon_{\mathrm{sn}}}{\sum_{n=1}^{N}\sum_{s=1}^{S_{n}}\varepsilon_{\mathrm{sn}}}$$

where *n* is the event index, *S_n_* indicates the steps taken by the primary and secondary protons in the voxel for the *n*‐th event, and *ε_sn_* and *L_sn_* are the energy deposited by proton and the mean energy loss per unit path length along the *s*‐th step in the *n*‐th event, respectively. To compute *L_sn_*, the ComputeElectronicDEDX() function of the G4EmCalculator class was used.^15^ From now on, the physical dose and *LET*_d_ originated from the collimator‐scattered protons will be referred to as *D* ^S^ and $=\pi r^{2}$ ${\mathrm{LET}}_{d}^{S}$, respectively, while those resulting from the unscattered protons and all protons (scattered + unscattered) will be indicated by the superscripts US and S + US, respectively.

### 2.C RBE calculation

The *RBE* was calculated using the linear–quadratic (LQ)‐based RBE model developed by McNamara et al.^21^:

(2) $$RBE\left[D,\frac{\alpha }{\beta },LET_{d}\right]=\frac{1}{2D}\left(\sqrt{{\left(\frac{\alpha }{\beta }\right)}^{2}+4D\left(\frac{\alpha }{\beta }\right)RBE_{\max}+4RBE_{\min}^{2}D^{2}}-\left(\frac{\alpha }{\beta }\right)\right)$$

where D is the physical dose, and *α* and *β* are the LQ parameters for the reference x‐ray radiation. The *LET*_d_ dependence is implicit in *RBE*_max_ and *RBE*_min_:

$$RBE_{\max}=0.99064+\frac{0.35605}{\left(\alpha /\beta \right)}LET_{d},RBE_{\min}=1.1012-0.0038703\times \sqrt{\frac{\alpha }{\beta }}LET_{d}.$$

### 2.D Evaluation

The *z*‐axis was the selected incident beam direction and its origin, *z* = 0, was located at the water surface; *x* and *y* were the transverse coordinates. We considered *z*_s_ = 5 mm as the representative normal tissue depth at the surface and *z*_c_ as the target center depth. The *x*‐position receiving the maximum dose by the collimator‐scattered protons at *z*_s_ was defined as *x*_s_ In this study, *α*/*β* was set to 10 Gy at the target center and to 3 Gy at *z*_s_.^22^ Note that these values fit for only some of the tumor sites. For the *α*/*β* parameter, a wide range of heterogeneity has been observed among tumor sites. In addition, recent literature review has revealed that the study heterogeneity for example, tumor stage, type of biological models and clinical endpoints gives large variation in the *α*/*β* parameter even for the same tumor site.^23^

The following parameters were evaluated for all the target geometries;

1. The maximum physical dose deposited by the collimator‐scattered protons at the depth *z*_s_ along the *x‐*axis, *D* ^S^(*x*_s_,0,*z*_s_), and at the center of the target, *D* ^S^(0,0,*z*_c_), are both normalized by the *D* ^US^ at the target center.
2. The dose‐averaged LET for the collimator‐scattered, unscattered, and all protons at (*x*_s_,0,*z*_s_), that is, $LET_{\text{d}}^{\text{S}}$ (*x*_s_,0,*z*_s_), $LET_{\text{d}}^{\text{US}}$ (*x*_s_,0,*z*_s_), and $LET_{d}^{\text{S}+\text{US}}$ (*x*_s_,0,*z*_s_), and at the target center, that is, $LET_{\text{d}}^{\text{S}}$ (0,0,*z*_c_), $LET_{\text{d}}^{\text{US}}$ (0,0,*z*_c_), and $LET_{d}^{\text{S}+\text{US}}$ (0,0,*z*_c_).
3. The relative biological effectiveness of the unscattered and all protons at (*x*_s_,0,*z*_s_), that is, *RBE* ^US ^(*x*_s_,0,*z*_s_) and *RBE* ^S+US^(*x*_s_,0,*z*_s_), and at the target center, that is,* RBE* ^US^(0,0,*z*_c_) and* RBE* ^S+US^(0,0,*z*_c_).

### 2.E Patient treatment plan

**Figure 3:**
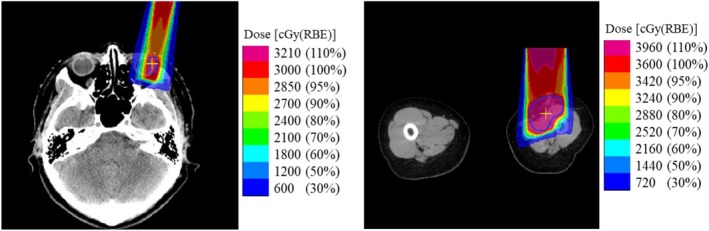
Dose distributions for Cases A, ocular melanoma, (left) and B, childhood rhabdomyosarcoma, (right).

Two simulated cases (Case A: ocular melanoma, Case B: childhood rhabdomyosarcoma) for which the collimator is beneficial to spare the surrounding normal organs were considered (Fig. 3). The treatment plan was created with the VQA TPS (Hitachi Ltd., Tokyo). A pencil beam algorithm, in which the lateral fluence profile is modeled as a double‐Gaussian function,^24^, ^25^ was used. The spot decomposition method was used to account for tissue heterogeneity and the collimator boundary across the beam's cross‐section.^26^, ^27^ The prescriptions were given to D99 and D50 of the clinical target volume (CTV), respectively, assuming that the *RBE* had a constant value of 1.1. The CTV size, range and FS of the targets, and the prescription per field are summarized in Table 2. The single field was used in both plans, and a 5‐mm collimator margin was selected to cover the CTV with the prescribed dose.

**Table 2:** Target geometry parameters and prescribed dose

Case	CTV size [ml]	Range [cm WEL]	Maximum SOBP width [cm WEL]	Field size (FS) [cm^2^]	Prescribed dose/fraction [Gy(*RBE*)]
A	12	2.4	2	1.3 × 1.5	6 (30/5)
B	139	5.0	4.5	4.4 × 11.8	1.8 (36/20)

CTV = clinical target volume, WEL = water equivalent length, SOBP = spread‐out Bragg peak

Note: The target FS was represented by the bounding rectangle of the target projected along the beam direction.

We evaluated the maximum dose increase nearby normal organs at the surface (lens in Case A and skin in Case B), the physical dose increase at the isocenter (set to the geometrical center of CTV), and the increases in *LET*_d_ and *RBE*. For Case B, the increase in RBE for the CTV was evaluated using *α*/*β *of 10 as well as 3 Gy following Mendonca et al.^28^

## 3 RESULTS

### 3.A Physical dose and *LET*_d_ distributions

**Figure 4:**
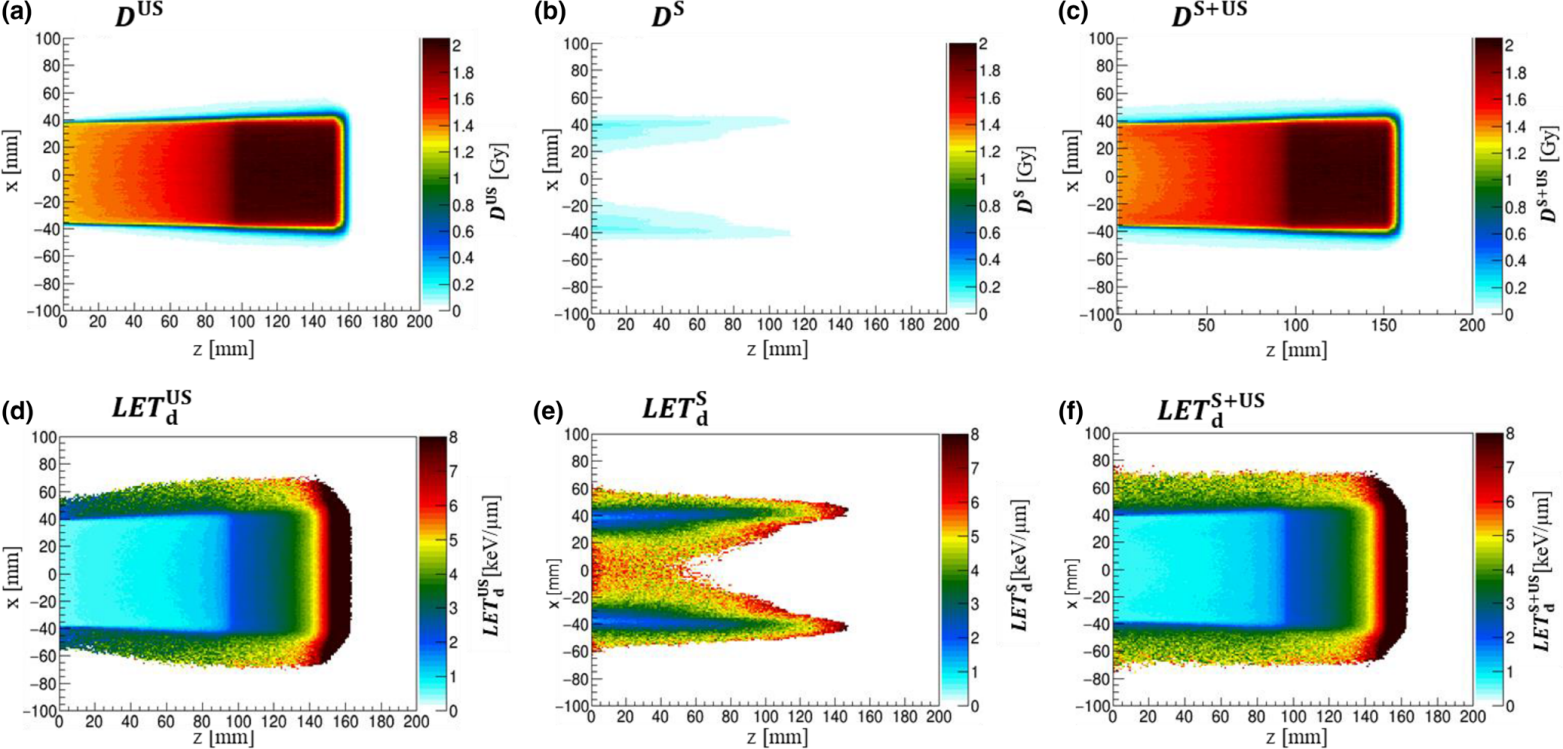
Distributions of the physical dose (*D*) and dose‐averaged linear energy transfer (*LET*_d_) in the *x‐z* plane, including the beam central axis for the target R15_S5_FS8, for the unscattered (superscript US), collimator‐scattered (superscript S), and all (superscript S + US) protons. The displayed regions are restricted to voxels with *D* ^S^ > 0.01 Gy.

Figures 4(a)–4(c) show the physical dose distributions in the *x–z* plane for unscattered, collimator‐scattered, and all protons for target R15_S5_FS8. Along the collimator edge, clear lines of the scattered dose can be observed: they exhibit the largest value at the surface and monotonically decrease with depth.

The *LET*_d_ distributions are shown, for the same categorized protons, in Figs. 4(d)–4(f); $LET_{\text{d}}^{\text{S}}$ exhibits larger values than $LET_{\text{d}}^{\text{US}}$ at the same location in water. At the water surface, the $LET_{\text{d}}^{\text{US}}$ distribution is nearly uniform along the *x‐*direction in field and slightly increases toward the out‐of‐field region, while the $LET_{\text{d}}^{\text{S}}$ one reaches its largest value (~8 keV/μm) at the field center, decreases to a quarter of it (~2 keV/μm) at the field boundary, and increases again toward the out‐of‐field region.

**Figure 5:**
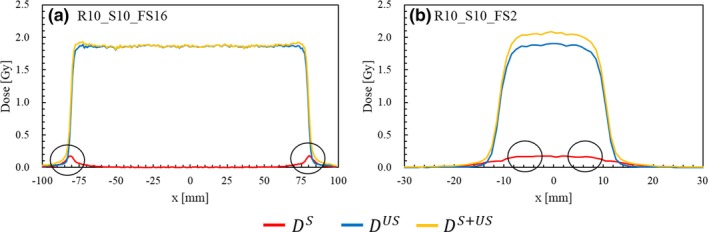
Distributions of the physical dose from the collimator‐scattered (*D* ^S^), unscattered (*D* ^US^), and all protons (*D* ^S+US^) at a 5‐mm depth along the *x*‐axis for the targets R10_S10_FS16 (a) and R10_S10_FS2 (b). The circled points indicate the *x*‐positions receiving the maximum dose by the scattered protons.

Figure 5 shows the *D* ^S^ distribution at a 5‐mm depth (*z* = *z*_s_) along the *x*‐axis for the targets R10_S10_FS16 (a) and R10_S10_FS2 (b), together with those of* D* ^US^ and *D* ^S+US^ for reference. Clear peaks can be observed at the collimator edge [Fig. 5(a)] but, as the FS is reduced, they overlap until they form a single large peak at the field center [Fig. 5(b)].

### 3.B Physical dose increase

**Figure 6:**
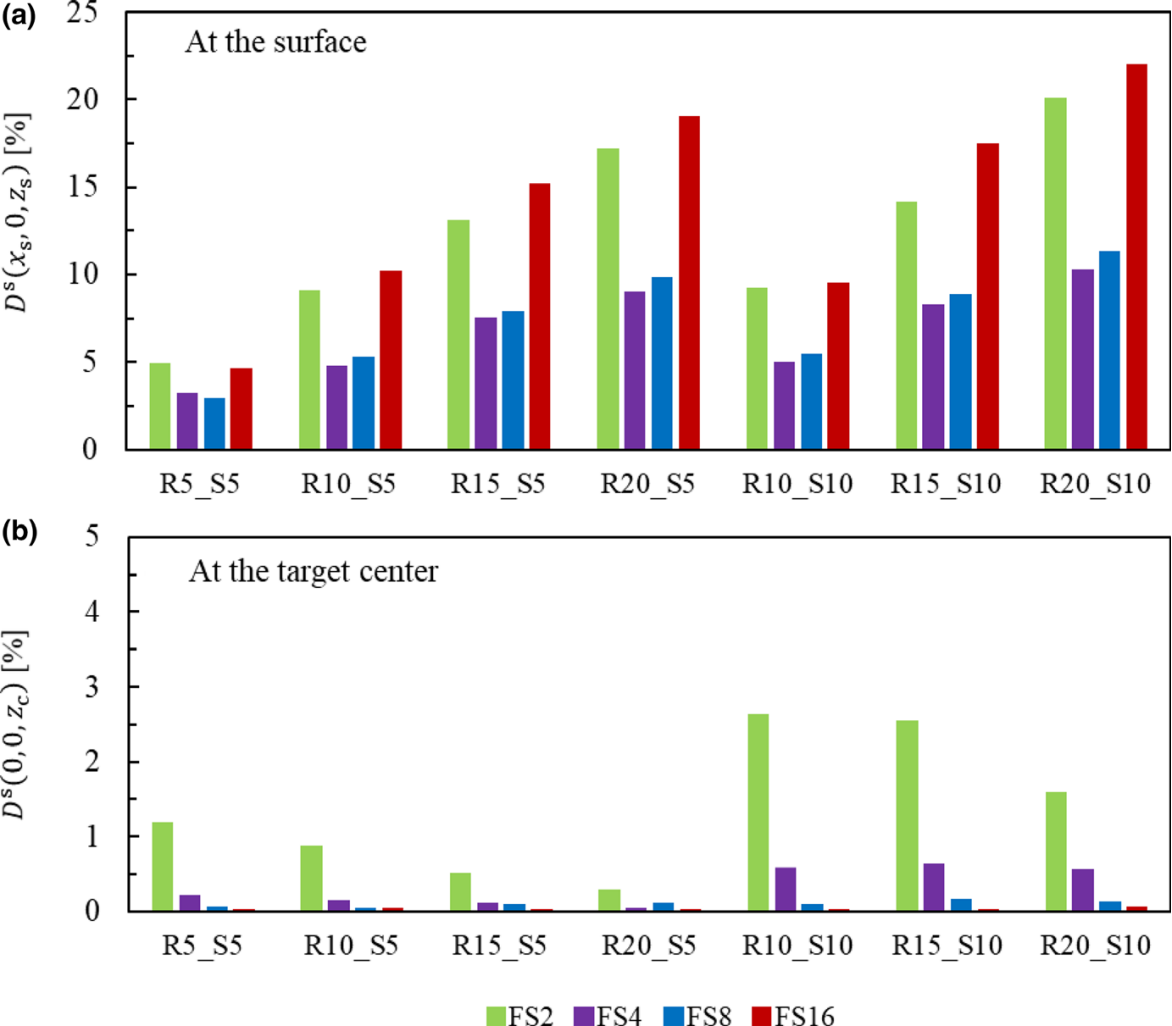
Maximum values of the physical dose from the collimator‐scattered protons (*D* ^S^) at a 5‐mm depth along the *x*‐axis (a) and at the target center (b), for the various target geometries listed in Table 1, normalized by the physical dose from the unscattered protons at the target center.

Figure 6(a) shows the maximum values of the scattered dose at a 5‐mm depth (*z* = *z*_s_) along the *x*‐axis for the various target geometries listed in Table 1. The maximum dose increase ranges from 3 to 22% over all tested conditions. The dosimetric impact increases with the range; it also increases with SOBP for ranges of 15 and 20 cm but remains almost unchanged for the 10 cm. The FS dependence is not monotonous; that is, at small and large FSs, the dose increase is larger compared to that at middle‐sized fields (FS = 4, 8) because, with a small FS, the overlapping scattered protons from several walls form the large central peak [see Fig. 5(b)]. In contrast, with a large FS, the beam angle with respect to the collimator wall increases, and so does the collimator scattering [see Fig. 5(a)]. The combination of these two effects results in the concave behavior against FS.

At the target center, the maximum scattered dose does not exceed 2.6% over all tested conditions, as shown in Fig. 6(b). It is largest with a small FS because of the largest overlap from the four collimator walls; for small FSs, the scatter dose decreases with the range because the scattered protons stop before reaching the target center.

### 3.C *LET*_d_ increase

**Figure 7:**
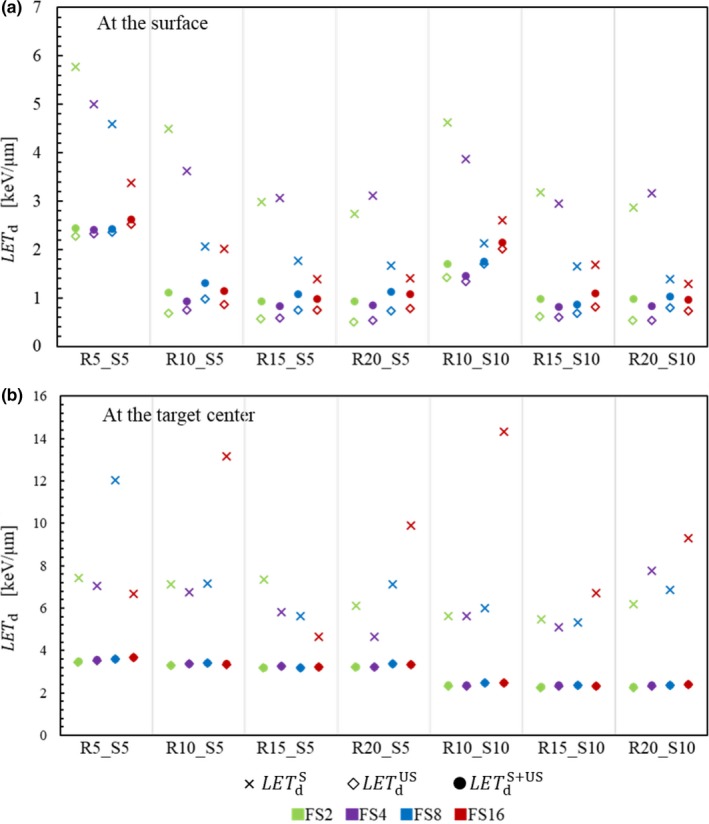
Dose‐averaged linear energy transfer from collimator‐scattered (*LET*_d_^S^), unscattered (*LET*_d_^US^), and all protons (*LET*_d_^S+US^) at a 5‐mm depth and at the x‐position receiving the maximum scattered dose from the collimator (a) and at the target center (b) for the various target geometries listed in Table 2.

Figure 7(a) shows the $LET_{\text{d}}^{\text{S}}$, $LET_{\text{d}}^{\text{US}}$, and $LET_{\text{d}}^{\text{S}+\text{US}}$ at a 5‐mm depth (*z* = *z*_s_) and at the *x*‐position receiving the maximum scattered dose from the collimator. As expected, the relationship $LET_{\text{d}}^{\text{S}}$ >** $LET_{\text{d}}^{\text{S}+\text{US}}$ > $LET_{\text{d}}^{\text{US}}$ holds in all tested targets, reflecting the protons' energy loss during scattering. However, although $LET_{\text{d}}^{\text{S}}$ can reach values 6.6 times greater than the maximum $LET_{\text{d}}^{\text{US}}$ at most, it does not largely influence the LET after the dose averaging: the maximum difference between $LET_{\text{d}}^{\text{S}+\text{US}}$ and $LET_{\text{d}}^{\text{US}}$ is 0.4 keV/μm.

Figure 7(b) shows the same quantities as Fig. 7(a) at the target centers. In this case, the collimator‐scattered dose is as small as shown in Fig. 6(b) (2.6% at most). Hence, even if $LET_{\text{d}}^{\text{S}}$ can reach large values as 14.3 keV/μm (target R10_S10_FS16), $LET_{\text{d}}^{\text{S}+\text{US}}$ and $LET_{\text{d}}^{\text{US}}$ are almost equal in all tested conditions (with a maximum difference of 0.08 keV/μm). In addition, both $LET_{\text{d}}^{\text{S}+\text{US}}$ and $LET_{\text{d}}^{\text{US}}$ are almost constant against the FS.

### 3.D RBE increase

**Figure 8:**
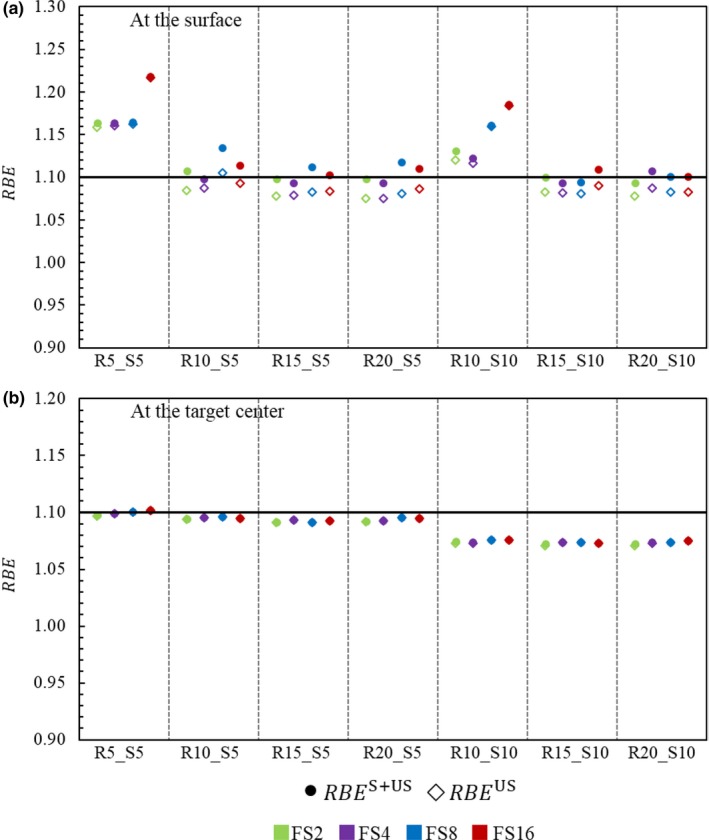
Relative biological effectiveness from the unscattered (*RBE* ^US^) and the scattered + unscattered protons (*RBE* ^S+US^) for the various target geometries listed in Table 2, at a 5‐mm depth, and at the *x*‐position receiving the maximum dose (*α*/*β* = 3 Gy) (a) and at the target center (*α*/*β* = 10 Gy) (b).

Figure 8(a) compares the *RBE* ^US^ and *RBE* ^S+US^ at a 5‐mm depth (*z* = *z*_s_) and at the *x*‐position receiving the maximum scattered dose from the collimator. Due to the increase in the *LET*_d_^S^ (see Fig. 7), *RBE* ^S+US^ is always greater than *RBE* ^US^; however, the magnitude of *RBE* increase is not significant because its maximum is only 0.04.

Figure 8(b) compares the same quantities at the target center. In this case, since *LET*_d_ does not change when the collimator‐scattered protons are included (see Fig. 7), the change in *RBE* is negligible.

### 3.E Physical dose, *LET*_d_, and RBE increase with the patient geometry

Table 3 shows the simulation results for the patient plans. The dose increase rate by the collimator scattering is described in terms of *D* ^US^ at the isocenter (5.5 Gy for Case A and 1.7 Gy for Case B). The closest target geometry of Case A among our tested conditions is R5_S5_FS2; the scattered dose is about 2.4% (Case A) and 5% (target R5_S5_FS2) at the surface. The patient plan has smaller scatter dose compared to the tested geometry mainly because both the range and SOBP are approximately 2 cm, which is smaller than the tested conditions. On the other hand, the Case B geometry is considered located between the targets R5_S5_FS4 and R5_S5_FS8. The water phantom simulation indicates a dose increase of about 3%, while a 4.2% increase is observed in Case B; this dose enhancement in patient geometry may be due to the overlap of the scattered doses from the sharp corners of the collimator and the skin location. The skin starts from the zero depth, where the scattered dose is maximum.

**Table 3:** The increases in physical dose (*D* ^S^), *LET*_d_, and *RBE* by the collimator scattering in OAR. *LET*_d_ and *RBE* were evaluated at the maximum *D* ^S^ point in OAR

Case	Maximum *D* ^S^ in OAR [Gy]	Maximum *D* ^S^ rate in OAR [%]	*LET*_d_ increase rate [%]	*RBE* increase rate [%]
*A*	0.13 (lens)	2.4	2.0	0.0
*B*	0.07 (skin)	4.2	8.2	0.9

OAR = organ at risk, LET_d_ = dose‐averaged linear energy transfer, RBE = relative biological effectiveness.

With regard to the *LET*_d_ and *RBE* increase, the changes from $LET_{\text{d}}^{\text{US}}$ to $LET_{\text{d}}^{\text{S + US}}$ and from *RBE* ^US^ to* RBE* ^S+US^ were calculated. The evaluation was done at the maximum dose point in the lens, for Case A, and skin, for Case B. In Case A, since the scattered dose is small, the *LET*_d_ increase is about 2.0% and the *RBE* does not increase at all; this result is slightly different from that of the target R5_S5_FS2, where the *LET*_d_ and *RBE* increase are 7.7% and 0.5%, respectively. As mentioned above, this difference is accounted for by the difference in range between the targets. In Case B, *LET*_d_ and *RBE* increase by 8.2% and 0.9%, respectively, while, in the targets R5_S5_FS4 and R5_S5_FS8, their increases are 2.8%–3.8% and 0.1%–0.2%, correspondingly. This difference may be attributed to the slight difference in the evaluation depth, as mentioned above. In both cases, we can observe a negligible increase in *RBE*, as expected from the simulations with test geometries.

For the target isocenter, the physical dose increase was 1.5% and less than 0.1% for Case A and B, respectively. No increase in RBE was observed in both cases, regardless of the *α*/*β* values (10 and 3 Gy).

## 4 DISCUSSION

We investigated the physical and biological impacts of collimator‐scattered protons used in a PBS system. In their pioneering work, van Luijk et al.^6^ focused on very small field sizes (up to 2 × 2 cm^2^), while this work was a systematic survey of FS up to 16 × 16 cm^2^, which covers a wider range of tumor sites and is almost the same size as that investigated by Titt et al.^7^ In the passive scattering, the impact of collimator scattering rapidly decreases as the FS increases from 3 × 3 to 10 × 10 cm^2^ and does not change from 10 × 10 to 15 × 15 cm^2^ (see Fig. 4 in Titt et al.^7^). We found that, in contrast to the passive scattering, the impact becomes large not only at small, but also large FSs, and this was attributed to the difference in proton beams' directionality between PBS and passive scattering. In passive scattering, the beam angle has a little correlation with the distance from the beam axis but, in PBS, the beam has a finite angle that is proportional to the lateral displacement with respect to the beam central axis. Since the X and Y scanning magnets are placed at different positions, the impact size is different among the scanned directions. Aside from the FS dependence, the physical dose impact by collimator‐scattered protons observed in this research is consistent (or, at least, is not in contrast) with the research of Titt et al.^7^ In both studies, the physical dose impact increases with the range at the water surface, according to the increased number of protons passing through the collimator; at the target center, the impact is comparable between different ranges and weakly increases with SOBP.

**Figure 9:**
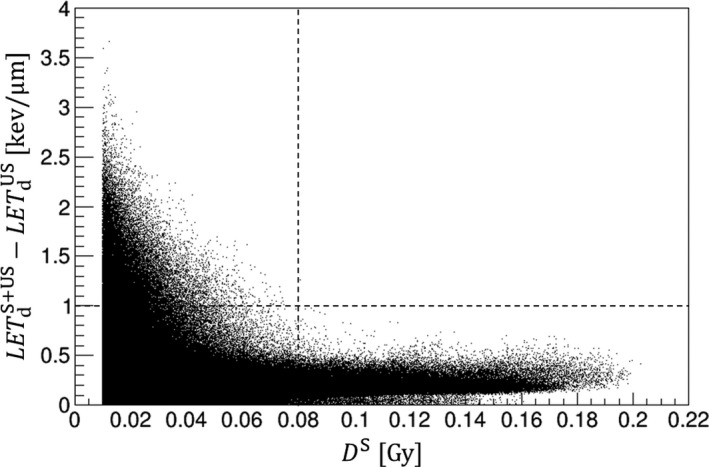
The increase in dose‐averaged linear energy transfer (*LET*_d_) against the increases in physical dose from the collimator‐scattered protons (*D* ^S^) for the target R15_FS8_S5, based on the voxels receiving more than 0.01 Gy.

To the best of our knowledge, this is the first study of the biological impact of collimator‐scattered protons using the LQ‐based LET‐dependent *RBE* model. In both water surface and target center depth, the $LET_{\text{d}}^{\text{S}}$ is highest at the field center and can exceed 10 keV/μm. However, the scatter dose is not large enough to increase the total *LET*_d_ by 1.0 keV/μm. Although we have reported results for only limited positions in each geometry, this is true for all the positions and geometries receiving a dose >0.3 Gy (15% of that at the target center). Figure 9 shows the scatter plot for the *LET*_d_ increase ($LET_{\text{d}}^{\text{S + US}}$ *−* $LET_{\text{d}}^{\text{US}}$) against the *D* ^S^ increase for the target R15_FS8_S5: all the voxels receiving a *D* ^S^ above 0.01 Gy are plotted. In general, an *LET*_d_ increase greater than 1 keV/μm is observed only in the voxels receiving small *D* ^S^ (<0.08 Gy, that is, 4% dose of the prescription); in those receiving a large *D* ^S^ (>0.08 Gy), instead, there is only a small increase (<1 keV/μm). This indicates that the enhanced $LET_{\text{d}}^{\text{S}}$ does not affect the total *LET*_d_, not only at the water surface and target center depth but at all positions.

One of the limitations of this work is that the number of scattered protons at the target center was not large enough to have statistically meaningful results. The large $LET_{\text{d}}^{\text{S}}$ variations observed for several geometries in Fig. 7 at FS ≥ 8 × 8 cm^2^ may be due to the statistical uncertainty. However, the input values for the *RBE* model ($LET_{\text{d}}^{\text{S + US}}$ and $LET_{\text{d}}^{\text{US}}$) were both calculated with a sufficient number of protons and gave reliable estimations.

We used the LQ‐based *RBE* model developed by McNamara et al.^21^ Due to the large variation in the *RBE* estimates because of the fundamental differences in experimental databases, model assumptions, and regression techniques,^29^ we estimated the model dependence of our results using two other models.^30^, ^31^ Different *RBE* models give different *RBE*; however, in both models, the magnitude of *RBE* increase is not significant (lower than 0.03 and 0.06 at the water surface and 0.003 and 0.005 at the target center, respectively).

The patient plan simulation results are almost consistent with the rectangular targets in the water phantom. However, due to the large variations of collimator shape and OAR location, the physical dose increase must be assessed patient‐by‐patient. Several analytical approaches have been proposed for passive scattering in the past^28^, ^32^, ^33^ to account for the collimator‐scattered protons in the TPS dose calculation engine. Several versions of fast Monte Carlo simulations^34^ have recently become available in commercial TPS, making it possible to evaluate the physical dose impact of collimator scattering in minutes with accuracy. Such simulation tools could enhance the PBS accuracy when using a collimator. On the other hand, when calculating *LET*_d_ and *RBE*, the scattered dose contribution can be neglected; analytical *LET*_d_ and *RBE* calculations^35^, ^36^ are promising methods for this purpose.

In this study, a patient‐specific collimator was used to effectively create a sharp penumbra along the outermost contour of the target. As an alternative, apertures that can adapt their shapes energy layer‐by‐energy layer have been developed,^2^, ^4^ but they may suffer from a greater collimator scattering in return for the increased conformity, which could be the subject of future investigations.

## 5 CONCLUSIONS

Both the physical and biological impacts of the collimator‐scattered protons used in PBS proton therapy were studied. Monte Carlo simulations revealed that a non‐negligible amount of physical dose was contaminated by the collimator scattering (3.0%–22.0% at the surface). The observed behavior was similar (or, at least, not contradictory) to previous research about the range and SOBP dependence in passive scattering. On the other hand, a different behavior was observed in terms of FS between PBS and passive scattering. The collimator‐scattered protons exhibited *LET*_d_ up to 6.6 times greater than the unscattered ones. However, when averaged by dose, the total *LET*_d_ was barely increased compared to the unscattered protons. Therefore, increased biological impact by the collimator‐scattered protons can be almost entirely attributed to an increased physical dose and not to the increase in RBE due to the *LET*_d_ increase.

## CONFLICT OF INTERESTS

We disclose that Shusuke Hirayama received funds from Hitachi, Ltd., Tokyo, Japan.

## Supporting information


**Fig S1** **.** Depth dose profiles along the beam central axis (a‐1, b‐1) and lateral profiles at a 5‐mm depth in water (a‐2, b‐2) for the targets R10_FS2_S10 (a‐1, a‐2) and R10_FS8_S10 (b‐1, b‐2), respectively.
