# Supplementary figures and images for: Physical and biological impacts of collimator‐scattered protons in spot‐scanning proton therapy
Source: J Appl Clin Med Phys. 2019 Jun 24;20(7):48–57. doi: 10.1002/acm2.12653 (PMC6612695; doi:10.1002/acm2.12653)

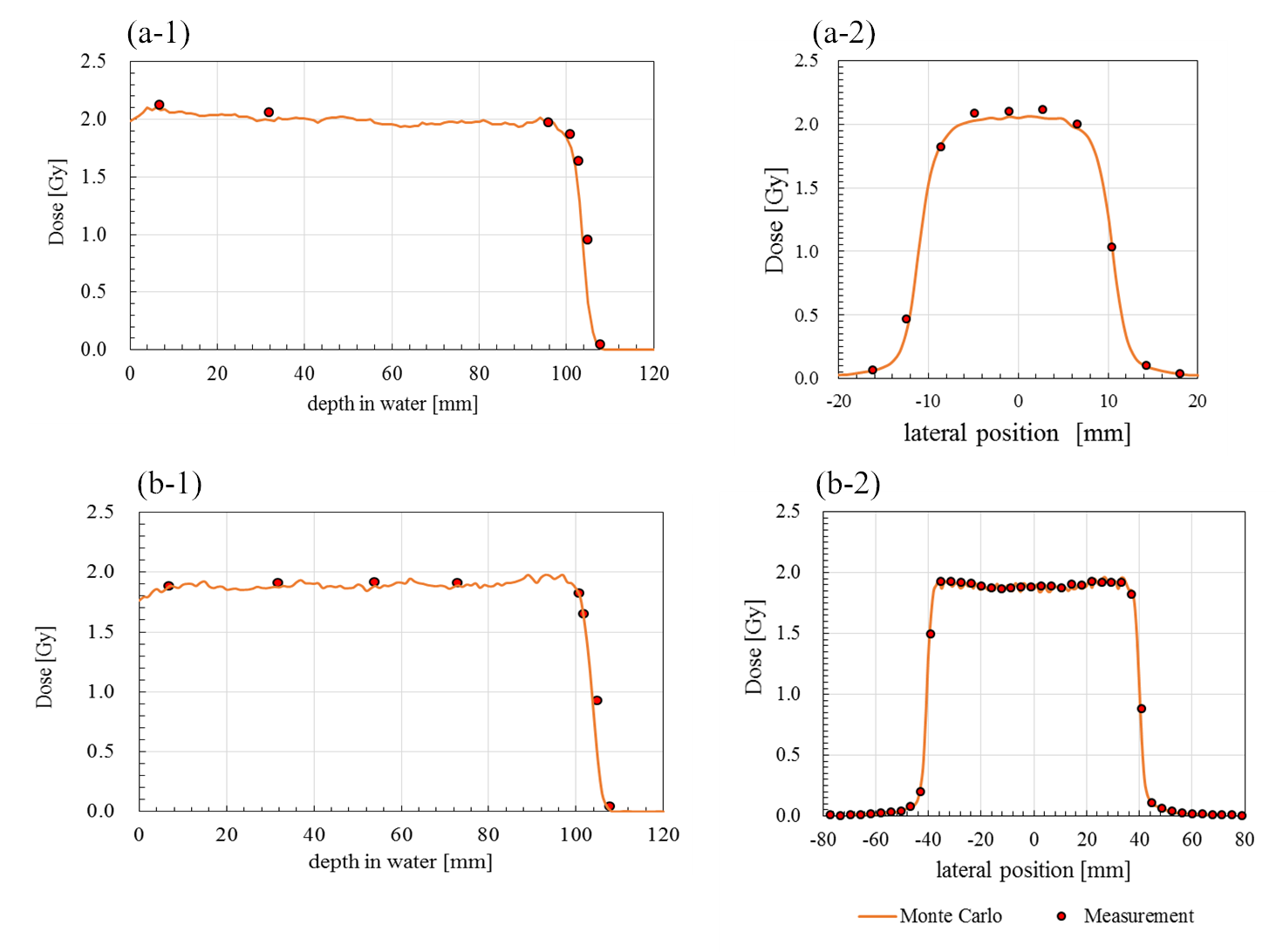

Supplement: Supplementary file 1 — Fig S1 . Depth dose profiles along the beam central axis (a‐1, b‐1) and lateral profiles at a 5‐mm depth in water (a‐2, b‐2) for the targets R10_FS2_S10 (a‐1, a‐2) and R10_FS8_S10 (b‐1, b‐2), respectively. [file ACM2-20-48-s001.tiff]
